# Broadening anticancer spectrum by preprocessing and treatment of T- lymphocytes expressed FcγRI and monoclonal antibodies for refractory cancers

**DOI:** 10.3389/fimmu.2024.1400177

**Published:** 2024-06-17

**Authors:** Lei Tang, Qinyi Sun, Mengyuan Li, Xiaoxiao Yu, Jinguo Meng, Yun Zhang, Yuxiao Ma, Aizhong Zeng, Zhuolan Li, Yuanyuan Liu, Xinyu Xu, Wei Guo

**Affiliations:** ^1^ Jiangsu Key Laboratory of Druggability of Biopharmaceuticals, State Key Laboratory of Natural Medicines, School of Life Science and Technology, China Pharmaceutical University, Nanjing, Jiangsu, China; ^2^ The Key Laboratory of Developmental Genes and Human Disease, Ministry of Education, The School of Life Science and Technology, Southeast University, Nanjing, Jiangsu, China; ^3^ Department of Research and Development, RegeneCore Biotech Co., Ltd, Nanjing, Jiangsu, China

**Keywords:** CAR-T, monoclonal antibodies, FcγRI, ADCC, solid tumors

## Abstract

**Background:**

Chimeric antigen receptor T (CAR-T) cell therapies have achieved remarkable success in the treatment of hematological tumors. However, given the distinct features of solid tumors, particularly heterogeneity, metabolic aggressiveness, and fewer immune cells in tumor microenvironment (TME), the practical utility of CAR-T cells for solid tumors remains as a challenging issue. Meanwhile, although anti-PD-1 monoclonal antibody (mAb) has shown clinical efficacy, most mAbs also show limited clinical benefits for solid tumors due mainly to the issues associated with the lack of immune cells in TME. Thus, the infiltration of targeted immunological active cells into TME could generate synergistic efficacy for mAbs.

**Methods:**

We present a combinational strategy for solid tumor treatment, which combines armored-T cells to express Fc-gamma receptor I (FcγRI) fragment on the surfaces for targeting various tumors with therapeutically useful mAbs. Choosing CD20 and HER-2 as the targets, we characterized the *in vitro* and *in vivo* efficacy and latent mechanism of the combination drug by using flow cytometry, ELISA and other methods.

**Results:**

The combination and preprocessing of armored T-cells with corresponding antibody of Rituximab and Pertuzumab exerted profound anti-tumor effects, which is demonstrated to be mediated by synergistically produced antibody-dependent cellular cytotoxicity (ADCC) effects. Meanwhile, mAb was able to carry armored-T cell by preprocessing for the infiltration to TME in cell derived xenograft (CDX) model.

**Conclusions:**

This combination strategy showed a significant increase of safety profiles from the reduction of antibody doses. More importantly, the present strategy could be a versatile tool for a broad spectrum of cancer treatment, with a simple pairing of engineered T cells and a conventional antibody.

## 1 Introduction

With the progress of medical technology, the contemporary medical field has gradually shifted from molecular therapy to cell therapy. Currently, 9 products have been approved by the Food and Drug Administration (FDA). CAR-T cell therapy has emerged as a promising approach in the treatment of hematological cancers (1, 2).

However, CAR-T research, even the whole field of cell therapy, still faces multiple challenges: limited effects on solid tumor, strong adverse effects, weak accessibility and high payment system (3–5). For solid tumor, due to its several characteristics, including their heterogeneity, toxicities, hostile tumor microenvironment (TME), and limited infiltration of immune cells (6), CAR-T cell therapy has been far less impressive in solid tumor. Although there are numerous popular targets, such as CD19, BCMA, CD20, HER-2, MSLN, and GD2, etc (7, 8). But, this requires redesigning each CAR molecule for each target, which leads to high initial research costs and slow progress in clinical application. Additionally, the FDA recently reported a safety announcement regarding CAR-T therapy’s potential to trigger T-cell lymphoma. Therefore, more in-depth research and new combination strategies are necessary to address these challenges and enhance the safety of cell therapy.

**Figure 1:**
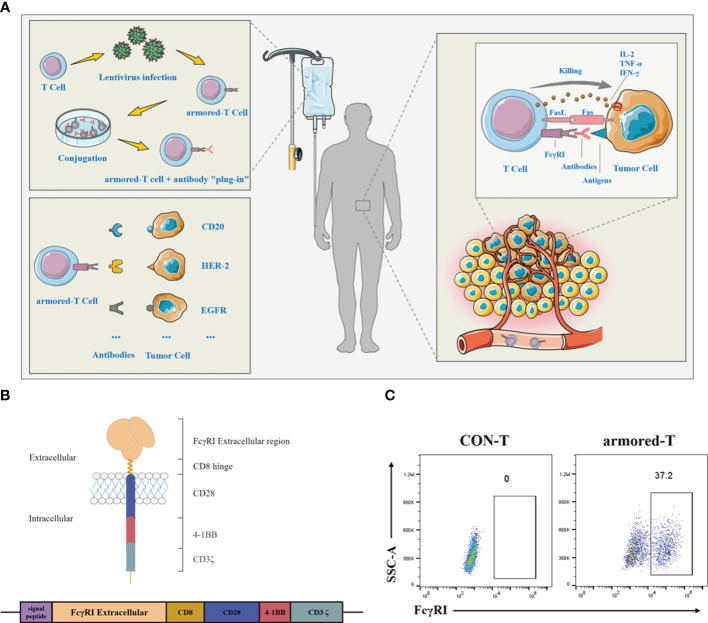
Molecular structure and characterization of armored-T cells *in vitro*. **(A)** Preparation, versatility and killing pathway of armored-T cells. Armored-T cells was prepared using lentiviral infection, followed by amplification and incubation with mAbs to form drug conjugates which were then transfused back into patients. There was no need to redesign the CAR molecule. The only need was to replace the mAb according to different indications, for effective killing of the tumor cells. Led by mAbs, Armored-T cells infiltrated tumor tissues and killed tumor cells through ADCC, Fas/FasL and cytokines. **(B)** Schematic representation of the chimeric FcγRI-CAR receptor constructs. The chimeric receptor comprised the extracellular domain of FcγRI, the hinge of CD8, the transmembrane domain of CD28, and two co-stimulatory domains containing CD28 and 4–1BB in tandem with the ζ-chain of the TCR/CD3 complex. **(C)** Expression of FcγRI-CAR in CD3^+^ human T cells, as assayed by staining with biotin-Fc and PE-conjugated streptavidin. The cells were analyzed using flow cytometry.

IgG Fc receptor I (FcγRI) is primarily expressed on the surfaces of monocytes, macrophages, dendritic cells, and other cell types, but not on T lymphocytes (9). It mediates antibody-dependent cellular cytotoxicity (ADCC) and cytokine release. Studies have shown that FcγRI exhibits the highest affinity for IgG1, when compared to other members of the FcγR family such as FcγRII and FcγRIII, with dissociation constants ranging from 10^−8^ to 10^−10^ mol/L (10). The binding of antibodies to IgG is a rapid process that typically occurs within minutes. Once activated, the binding of antibodies to FcγRI can become more stable, leading to a series of biological effects (11). It has been demonstrated that IgG binding to FcγRI is higher under acidic conditions compared to physiological pH levels (12–14). This property also enables drugs to be more effective in tumor tissue sites while avoiding toxicity to normal tissues (15). Based on the above, we designed a novel molecule, which can be expressed on armored-T cell and bind to approved antibody drug. Armored-T cells surface expressed an FcγRI fragment which may bridge with different antibody with IgG1 Fc targeting infiltration to TME to achieve universal therapy (**Figure 1A**).

In this study, we selected CD20 and HER-2 as targets to demonstrate whether the combination drug can kill target tumor cells *in vitro* and *in vivo* compared to single antibody treatment. These findings provide preliminary evidence for the feasibility of the combination strategy. The strategy not only provided functional immune cells to enhance the efficacy of antibodies by enabling T cells to perform ADCC, but also endowed them with enhanced tumor killing potential and prolonged activity through genetic editing. Moreover, we eliminate the need for designing new CAR molecules for different disease types, thereby establishing a foundation for treatment of a broad spectrum of cancers.

## 2 Materials and methods

### 2.1 Cell lines

The HEK293T, MCF-10A, SK-BR-3, SK-OV-3, MDA-MB-231, HCT-116, Sgc7901 and MKN-45 were cultured in DMEM (Gibco) supplemented with 10% fetal bovine serum (FBS; Gibco), 2 mM L-glutamine (Gibco), 0.1 mg/mL streptomycin (Gibco), and 100 U/mL penicillin (Aladdin). Hematological malignancy cell line Jurkat76, Daudi, Raji and K562 were maintained in RPMI-1640 (Gibco) supplemented with 10% FBS, 2 mM L-glutamine, 0.1 mg/mL streptomycin and 100 U/mL penicillin. Peripheral blood mononuclear cells (PBMC) obtained from healthy volunteers were cultured in X-VIVO™15 medium (Lonza Group Ltd) supplemented with 100 U/mL penicillin and 0.1 mg/mL streptomycin. All of the above cells were purchased from Wuhan Pricella Biotechnology Co. Ltd. In addition, Raji cells with luciferase were donated by Nanjing Regenecore Biotechnology Co. Ltd., while other tumor cell lines featuring luciferase were prepared in-house by our laboratory.

### 2.2 Vector construction, lentivirus production, and gene transduction

In the construction of armored-T vectors, the extracellular domain of human FcγRI from a PBMC cDNA library was amplified using PCR (Thermo Fisher Scientific). The armored-T vectors were generated by fusing FcγRI to the hinge region of CD8α. The modified molecule was constructed with the signal peptide, extracellular domain of FcγRI, the hinge and transmembrane domains of human CD8α; intracellular domains of human CD28 and 4–1BB, and the intracellular domain of human CD3ζ. The specific sequence came from a patent which we applied for, in conjunction with our partner company (Patent Number: CN201910500433.6).

The lentiviral supernatant was obtained from HEK293T cells and three-plasmid system comprising armored-T lentiviral vector plasmid, pMD2.G and psPAX2. The lentiviral supernatant was harvested 48, 72 and 96 hours after transfection and were centrifuged at 80000 g to remove cell debris, and the resultant fluids were kept frozen at -80°C. The transduction efficiency of lentivirus was determined using flow cytometry.

To generate armored-T cells, CD3^+^ T lymphocytes were selected from PBMCs using MACS^®^ Technology (Miltenyi Biotech) and were activated with T Cell TransAct (Miltenyi Biotech), 200 U/mL hIL-2 (Pepro Tech), 10 ng/mL hIL-7 (Pepro Tech), and 5 ng/mL hIL-15 (Pepro Tech). After 48 hours of activation, the T cells were transduced with lentivirus along with polybrene (6 μg/mL; Sigma-Aldrich) in half of the medium. After centrifugation at 800 g for 1 hour at 25°C, the cells were cultured in an incubator at 37°C for 6 hours. The medium was supplemented to normal volume with the same amount of cytokines were added, and the cells still cultured in the incubator at 37°C. After three days, the transduction efficiency was determined using flow cytometry.

### 2.3 Erythrocyte rosette test

The erythrocyte Rosette test was used to identify armored-T cells directed to tumor cells by mAb. The Raji, K562 and Daudi cells were plated in 24-well plates, each at a density of 2×10^4^ cells/well and incubated with armored-T cells or CON-T cells (E:T=3:1). Then, the cells were incubated with Rituximab (10 μg/mL; Roche) at 37°C in a 5% CO_2_ atmosphere. Cells that were not incubated with Rituximab as control. The cells were photographed under a microscope (Olympus) equipped with a digital camera.

### 2.4 Assays for antibody binding, cell activation, and cell proliferation

To measure the antibody-binding capacity of chimeric receptors, different positive rates of armored-J76 cells were obtained by transfecting J76 cells with different MOI values (MOIs of 1, 5, 10, 20 and 30) using lentivirus. Then, 5×10^5^ armored-J76 cells were seeded in a 24-well plate with FITC-Rituximab or PE-Pertuzumab (10 mg/mL; Sino Biological Inc) and incubated in the dark for 1 hour at 37°C in a 5% CO_2_ atmosphere. After washing twice with PBS, cell staining was measured using a flow cytometer.

The levels of cell surface activation marker CD69 and the cell degranulation marker CD107a were determined as the degree of activation of the cells. Armored-T cells and target cells were seeded in 96-well plates (E:T=3:1) and placed in a 5%-CO_2_ incubator at 37°C. After culturing for 16 hours, CD69 level was determined using flow cytometry. The assay for CD107a was done after blocking its internalization (endocytosis) by the addition of monensin 1 hour after the seeding. This was followed by further incubation for 6 hours, after which CD107a was determined using flow cytometry.

### 2.5 *In vitro* tumor cell lysis assay

The cytolytic activity of T cells was measured with standard L-lactate dehydrogenase (LDH)-release assay, flow cytometry and luciferase reporter assay. The LDH assay was used to determine the efficiency of lysis of solid tumor cell lines after co-culturing with armored-T cells. The target cells were seeded in a 96-well plate at a density of 1×10^4^ cells/well and incubated with armored-T cells (E:T=1:1, 1.5:1, 3:1) in the presence of Pertuzumab (10 μg/mL; Roche). The process followed the instructions of the LDH Cytotoxicity Assay Kit (Beyotime). The percentage of specific lysis was calculated as below:


$$\begin{array}{c} Cytotoxicity\;\left(\%\right)\;=\;\left(exp.value\;-\;low\;control\right)\;/\;\\ \left(high\;control\;-\;low\;control\right)\;\times 100. \end{array}$$

In the determination of T cell toxicity on hematoma cells, the proliferation of the tumor cells was inhibited using the same method as solid tumors. Then, the tumor cells were labeled with CellTrace™ Far Red Cell Proliferation Kit (Invitrogen, USA) and seeded in a 96-well plate at same E:T ratio with or without Rituximab (10 μg/mL). After incubation for 16 hours at 37°C in 5% CO_2_ atmosphere, the cells were stained with propidium iodide solution in the dark for 15 minutes, and specific lysis was determined using flow cytometry.


$$\begin{array}{c} Cytotoxicity\;\left(\%\right)\;=(PI^{+}number\;of\;tumor\;cells)/\;\\ \left(total\;number\;of\;tumor\;cells\right)\;\times \;100 \end{array}$$

Luciferase-expressing tumor cells were seeded in a white-walled 96-well plate at a density of 1×10^4^ cells/well and incubated with armored-T or CON-T cells (E:T=1:1) in the presence of mAbs (0/10 μg/mL). Tumor cell viability was monitored 24 hours later by Bright-Glo™ Luciferase Assay System (Promega, USA). Emitted light was measured with a luminescence plate reader, and cytotoxicity was calculated using the following formula:


$$\begin{array}{c} Cytotoxicity\;\left(\%\right)\;=\;(1-\left(RLU\;cocultured\;sample\right)/\\ \left(RLU\;cancer\;cells\;only\right))\times \;100 \end{array}$$

### 2.6 Assay of cytokine release

In the determination of cytokine release, 1×10^4^ solid cells or 2×10^4^ hematoma cells were seeded in a 96-well plate and incubated with armored-T cells or CON-T cells (E:T=3:1), with addition of Pertuzumab or Rituzumab (10 μg/mL). After 24 hours, the levels of IFN-γ, TNF and IL-2 in the culture supernatants were measured using ELISA kit (Thermo Fisher Scientific).

### 2.7 Flow cytometry

The antibodies used for flow cytometric analysis are shown in **Supplementary Table 2**. The cells were analyzed using a Gallios Flow Cytometer (BD Biosciences). Data analysis was performed using Flow Jo Version 7.2.2 software (Tree Star).

### 2.8 *In vivo* antitumor effect in a mouse model of B-cell lymphoma

All *in vivo* experiments were conducted in line with the guidelines of the Institutional Animal Care and Use Committee of China Pharmaceutical University (Nanjing, China). To determine the *in vivo* antitumor effect of armored-T cells, six-week-old NCG female mice (Model Animal Research Center, Nanjing University) were injected with 5×10^5^ luciferase-expressing Raji cells through the tail veins. When the fluorescence intensity in *in vivo* imaging reached 5×10^5^ p/s, the mice were randomly divided into 4 groups and treatment was started: control group given PBS; Rituximab group given Rituximab at a dose of 30 mg/kg; armored-T cell group given the cells at a dose of 3×10^6^ armored-T cells per mouse, and combination group co-administered armored-T cells + Rituximab (3×10^6^ cells/mouse and 30 mg/kg). All treatments were administered via caudal intravenous injection for 2 weeks except the armored-T cells were injected only once.

Tumor engraftment and growth were measured using a Xenogen IVIS-200 system (Caliper Life Sciences). Imaging commenced 5 minutes after intraperitoneal injection of D-luciferin potassium salt at a dose of 3 mg/mouse. The photons were quantified using the Living Image 3.0 software. After the experiment, peripheral blood and spleen were taken. The mice were sacrificed before the fluorescence intensity of tumor cells reached 1×10^10^ p/s.

### 2.9 *In vivo* antitumor effect in a xenografted mouse

Six-week-old NCG female mice were subcutaneously inoculated above their right flanks with 5×10^6^ SK-OV-3 cells. The size of tumor was measured twice a week using calipers. When the tumors reached sizes of 200 - 300 mm^3^, the mice were divided into 3 groups and treated with PBS, Pertuzumab (15 mg/kg, i.v., double the first dose), or combination of armored-T cells and Pertuzumab (2×10^6^ cells/mouse and 15 mg/kg, i.v., double the first dose) via tail vein injection once a week for four consecutive weeks. During the treatment period, changes in mouse body weight and tumor volume were measured and analyzed. After the experiment, peripheral blood and tumor tissues were taken from each mouse. The mice were sacrificed before the average tumor size reached 2000 mm^3^.

### 2.10 Statistical analysis

All data are the results of at least 3 independent experiments. Each experimental group consisted of at least 5 mice. All statistical analyses and graph preparations were performed with GraphPad Prism 9. Results are expressed as mean ± standard deviation (SD) or mean ± standard error of the mean (SEM). Two-sample comparisons were done using two-tailed Student’s *t*-test, while multi-group comparisons were carried out with two-way ANOVA and Dunnett’s multiple comparison test. The survival curves of mice were analyzed using the Log-rank test method. Values of *p*< 0.05 were considered indicative of statistically significant differences.

## 3 Results

### 3.1 Expression of the FcγRI receptor on primary T cells

The FcγRI is a receptor with high affinity for IgG Fc protein. In this study, we acquired the sequence of FcγRI (Patent Number: 201910500433.6) and combined it with the hinges of CD8, CD28 transmembrane domain, and CD28 and 4–1BB intracellular co-stimulatory domains in tandem with CD3ζ signaling domain (**Figure 1B**). Thereafter, T cells were transduced with FcγRI-28-BB-ζ lentivirus. Results showed that T cells maintained stable FcγRI-28-BB-ζ ratios at approximately 37.2% for two weeks (**Figure 1C**; **Supplementary Figure 1A**). Moreover, the transduction using lentivirus did not change the ratio of CD4 and CD8 in T cells (**Supplementary Figure 1B**).

### 3.2 Binding capacity of FcγRI molecule to IgG mAbs and optimal saturation concentration

**Figure 2:**
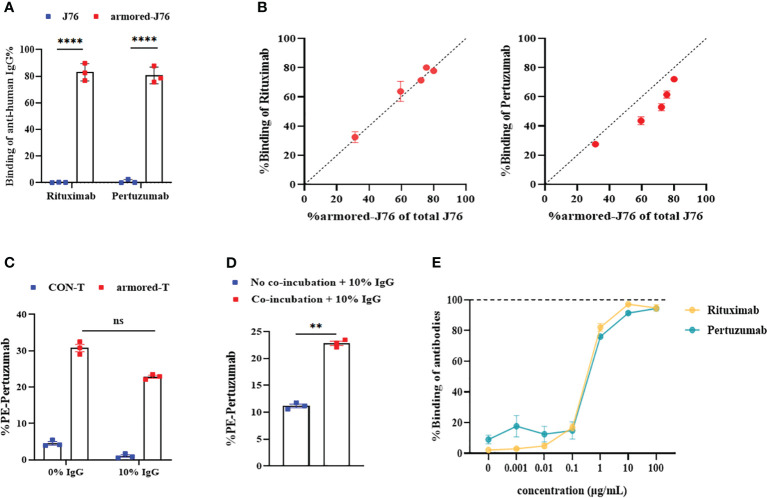
Binding capacity of FcγRI molecule to IgG mAbs and optimal saturation concentration. **(A)** The binding efficiencies of Rituximab and Pertuzumab after FcγRI-J76/J76 cells were co-cultured with the antibody drugs for 1 hour at room temperature, as determined using flow cytometry. **(B)** Flow cytometric analysis of the relationship between the expression abundance of FcγRI and the binding capacity of FcγRI molecules to the antibody drugs at different MOIs using PE-conjugated Pertuzumab or FITC-conjugated Rituximab. **(C)** The binding capacities of the antibodies after armored-T cells and antibody drugs were co-cultured at 37°C for 1 hour in the presence or absence of 10% plasma, as measured using flow cytometry. **(D)** The binding capacity of T cells to antibodies, as measured using flow cytometry after armored-T cells and antibody drugs were co-cultured or cultured separately at 37°C for 1 hour and then added to a system containing 10% human plasma. **(E)** Levels of binding of each of the antibody drugs when T cells were co-cultured with FITC-conjugated Rituximab and PE-conjugated Pertuzumab at various antibody concentrations at 37°C for 1 hour. Flow cytometry was used to determine levels of binding. Graphs show data as mean ± SD. Statistical significance was calculated using Student *t*-test for **(D)**, and two-way ANOVA for multiple comparisons for **(A, C)**. ns(no significance) p>0.05; **p< 0.01;****p< 0.0001. Error bars represent standard error of the mean.

To investigate the binding capacities of FcγRI and IgG mAb, we expressed FcγRI on J76 (**Supplementary Figures 2A, B**), and co-incubated it with Rituximab and Pertuzumab at room temperature for 1 hour. The binding rates of antibodies to FcγRI were determined. Relative to WT-J76 cells, the binding rates of Rituximab and Pertuzumab to FcγRI were around 83.0% and 80.6% (**Figure 2A**; **Supplementary Figures 2C, D**), respectively, indicating no significant difference in the capacity of FcγRI to bind to different antibodies. To study the relationship between the abundance of FcγRI and the binding affinity of antibodies, we expressed different amounts of FcγRI on the surface of J76 cells. Through co-incubation with Rituximab or Pertuzumab, it was found that the expression level was directly proportional to the binding capacity (**Figure 2B**).

Despite the proven strong binding between armored-T cells and IgG, human plasma contains 75%-80% immunoglobulins which have compositions similar to IgG. We simulated changes of binding efficiency between armored-T cells and Pertuzumab in the presence or absence of 10% human plasma to prevent competitive binding between immunoglobulins and armored-T cells. It was revealed that the binding efficiency between armored-T cells and Pertuzumab decreased from 30% to 22% under the condition of 10% plasma (**Figure 2C**), indicating a slight degree of influence. Therefore, we attempted pre-incubation of armored-T cells with mAbs to weaken the competitive binding of IgG in serum. A comparison of co-incubation or non-incubation of armored-T cells with Pertuzumab revealed that co-incubation at room temperature for 1 hour resulted in a significantly higher binding efficiency (**Figure 2D**). Then, we determined the optimal proportion for co-incubation for subsequent *in vivo* administrations and dosages of the combination therapy. So we controlled the level of FcγRI and added gradient concentrations of antibody to fixed amounts of armored-T cells. It was found that when 5×10^4^ cells were co-incubated with Rituximab or Pertuzumab at a concentration of 10 μg/mL, this ratio resulted in binding efficiency close to saturation. Thus, it was used in subsequent *in vivo* drug administrations (**Figure 2E**).

### 3.3 T lymphocytes expressing FcγRI-28-BB-ζ enhanced *in vitro* cytotoxic efficiency

**Figure 3:**
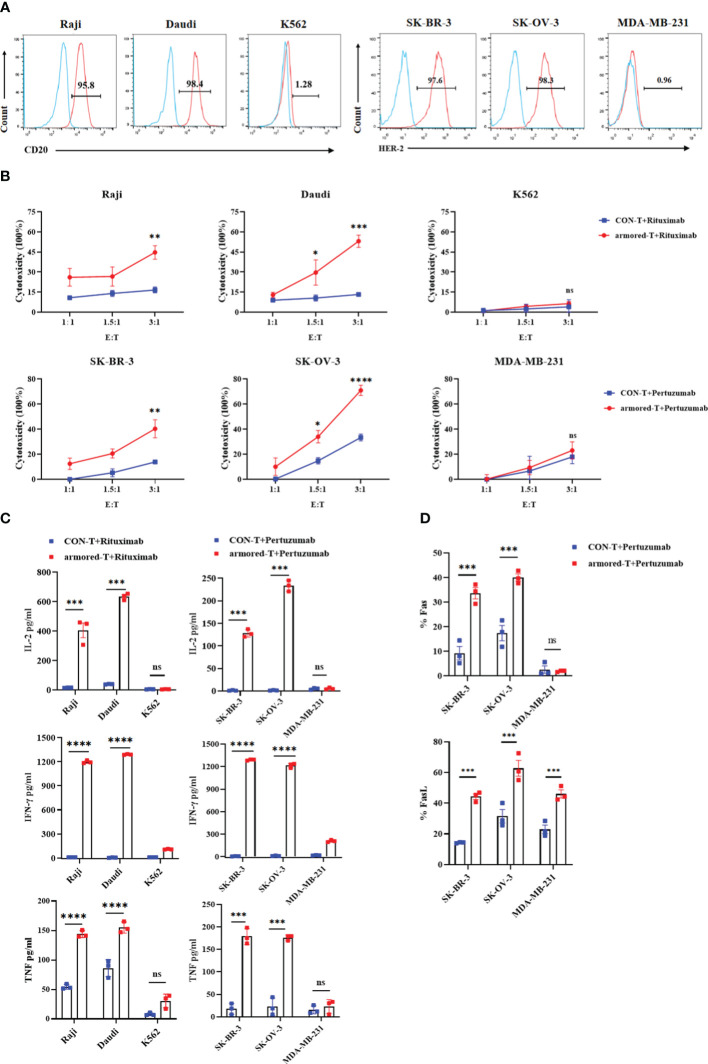
armored-T cells in combination with IgG antibody drugs exhibited enhanced cytotoxic activity and cytokine secretion against tumor cells. **(A)** Surface expression of CD20 and HER-2 on tumor cells, as determined using flow cytometry. **(B)** Cytotoxicity of hematologic tumors, as evaluated using CellTrace™ Far Red Cell Proliferation Kit and PI flow cytometric assay. Target cells were labeled with CellTrace™ Far Red Cell Proliferation Kit, and after co-incubation of armored-T cells/CON-T cells (E:T=1:1, 1.5:1, 3:1) with Rituximab (10 g/mL) for 16 hours, the target cells were stained with PI and analyzed flow cytometrically. Solid tumors were assessed using LDH release assay. Tumor cells were co-incubated with armored-T cells (E:T=1:1, 1.5:1, 3:1) and Pertuzumab (10 μg/mL) for 4 hours, and the levels of released LDH were measured via peak absorption at a wavelength of 490nm. **(C)** The levels of IL-2, IFN-γ and TNF released in the cell supernatants collected after 24 hours of co-incubation of each antibody drug (10 μg/mL), armored-T cells or CON-T cells, and tumor cells (E:T=3:1), as measured using ELISA assay kit. **(D)** The expressions of Fas and FasL in cells, as determined using flow cytometric analysis. Statistical significance was calculated using two-way ANOVA for multiple comparisons. ns(no significance) p>0.05;*p<0.05;**p<0.01; ***p< 0.001;****p< 0.0001. Error bars represent standard error of the mean.

The efficiency of armored-T cells combined with different antibodies in lysing tumor cells was investigated *in vitro* using several targets, among which were CD20, HER-2 and EGFR. Firstly, the tumor cells with surface markers were determined using flow cytometry (**Figure 3A**). The tumor cell lines without targets were used as control cells, while the human mammary fibroblast cells MCF-10A served as irrelevant cells for determination of toxicity to normal tissues. We studied changes in the anti-tumor effect after the combination of armored-T cells with IgG antibodies. After co-incubation of armored-T cells with Rituximab and CD20+/CD20- tumor cells at room temperature for 4 hours, it was observed that aggregation was formed between armored-T cells and Raji and Daudi, while K562 and CON-T cells did not exhibit any aggregation (**Supplementary Figure 3A**). This suggested that IgG may act as a bridge for connecting armored-T with tumor cells.

Hematological tumor cells were labeled with CellTraceTM Far Red Cell Proliferation Kit and co-cultured with armored-T cells or CON-T cells at E:T (the effector cells refer to the cells expressing FcγRI) ratio of 1:1, 1.5:1, 3:1. After incubating for 16 hours with or without the addition of Rituximab, the percentage apoptosis of target cells was measured flow-cytometrically. The results showed that the killing efficiencies of Raji and Daudi cells were significantly higher than those of control group treated with CON-T cells and antibody drugs (**Figure 3B**). As expected, the killing effect of armored-T+Rituximab group on K562 cells was minimal. The solid tumor cells were co-incubated with tumor cells and armored-T cells under the same co-incubation conditions, and the cytotoxic capacity was determined after 4 hours. The results showed that the killing efficiencies of SK-BR-3 and SK-OV-3 with positive HER-2 expression were 78.45% and 31.8% (E:T=3:1), respectively (**Figure 3B**). There were no significant differences between the two groups of MDA-MB-231 cells, and the killing efficiency was lower than 25%. In addition, we explored that the killing effect is produced by armored-T cells under the guidance of mAbs. The results showed that among the positive cell lines, the group treated with armored-T cells in conjunction with mAbs exclusively demonstrated a significant cytotoxic effect, markedly distinguishing themselves from the other three groups (**Supplementary Figures 3B, C**).

We also assayed the expressions of cytokines related to tumor cell apoptosis. The armored-T cells or CON-T cells were co-cultured with tumor cells at antibody level of 10 μg/mL for 24 hours. The supernatants were assayed for secretion levels of IL-2, IFN-γ and TNF. The expression levels of IL-2, IFN-γ and TNF in armored-T cells were significantly higher than those in CON-T cells (**Figure 3C**). The killing effect and cytokine release of armored-T cells with other mAbs was consistent with those of CD20 and HER-2 (**Supplementary Figure 4**). These results indicate that antibodies enhanced the activation, proliferation and cytotoxic capacity of armored-T cells. Moreover, the LDH data of MCF-10A showed that armored-T cells had no effect on normal tissue cells (**Supplementary Figure 3D**).

### 3.4 Mechanism underlying the tumor cell-killing potential of armored-T cells conjugated with antibody

The mechanism through which T cells kill tumor cells involves direct killing and inducing apoptosis. The former induces T cells to secrete mediators such as perforin and Granzyme B, while the latter up-regulates FasL on T cells and Fas on tumor cells. We determined FasL expression in T cells and Fas expression in tumor cells under the same co-culture conditions. The results showed that after co-incubation with Rituximab, the expressions of Fas in Raji and Daudi cells, and the expressions of FasL in armored-T cells were significantly higher than those in CON-T cells, while the expressions in K562 cells were basically unchanged (**Figure 3D**; **Supplementary Figure 3E**). The HER-2+/HER-2- tumor cell lines showed the same trend, indicating that armored-T cells bound to antibody enhanced the killing effect on tumor cells more obviously by activating the Fas/FasL pathway.

### 3.5 Binding of IgG to FcγRI-28-BB-ζ induced T-cell activation and proliferation

**Figure 4:**
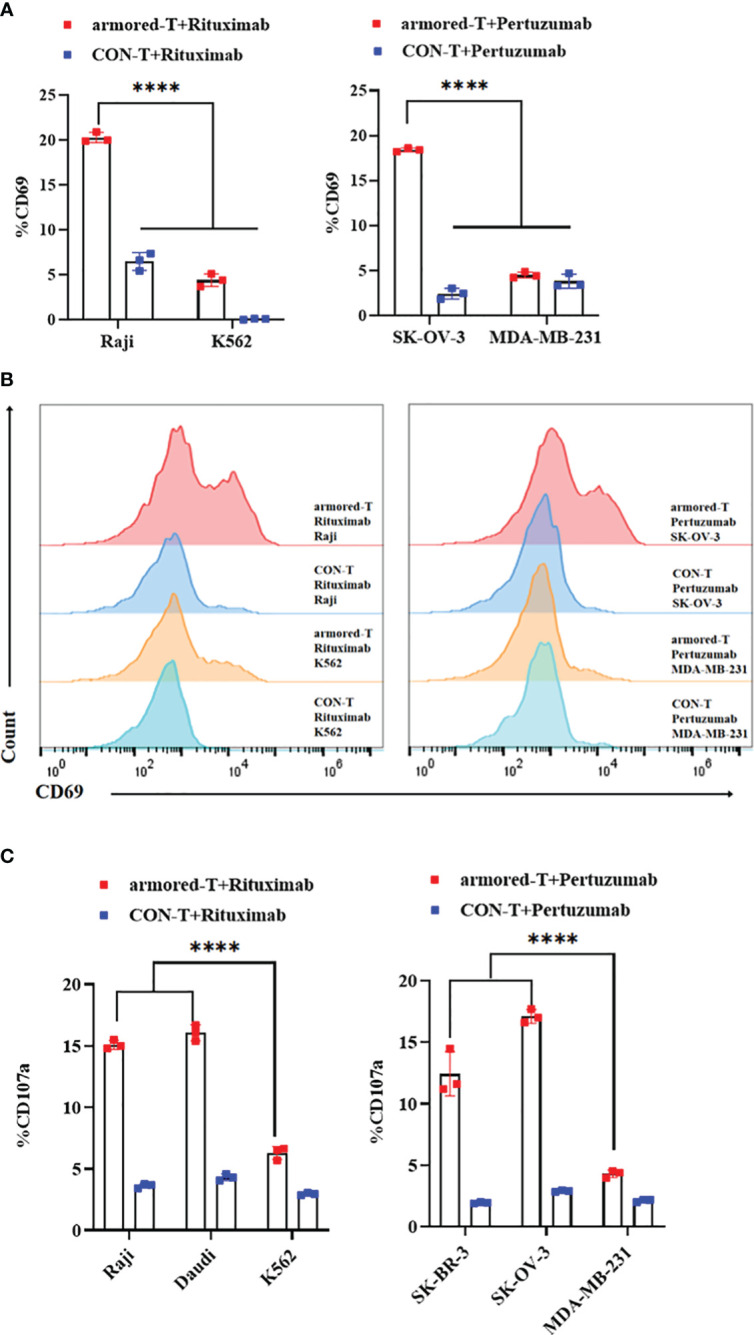
armored-T cells in combination with IgG antibody drugs significantly enhanced the activation of T cells. **(A, B)** Armored-T cells/CON-T cells were co-cultured with target cells (E:T= 3:1) in the presence of antibody at a concentration of 10 μg/mL for16 hours, and analyzed for CD69 expression using flow cytometry. **(C)** Armored-T cells/CON-T cells were co-cultured with target cells (E:T= 3:1) in the presence of antibody at a concentration of 10 μg/mL for 6 hours, after which CD107a was measured. Statistical significance was calculated using two-way ANOVA for multiple comparisons. *P< 0.05; **p< 0.01; ***p< 0.001; ****p< 0.0001. Error bars represent standard error of the mean.

The activation, degranulation and proliferation of armored-T cells after combination with antibody drugs and tumor cells were investigated by determining the expression of CD69 and CD107a. We compared armored-T cells and CON-T cells incubated with positive or negative tumor cells at an effector-target ratio of 3:1 at antibody concentration of 10 μg/mL. The release of CD69 (**Figures 4A, B**; **Supplementary Figure 5A**) and CD107a (**Figure 4C**) in the armored-T cells was significantly increased, when compared with the CON-T cells, indicating that the activation potential of armored-T cells was higher when stimulated with tumor cells and antibody than that of CON-T cells. However, in MDA-MB-231 and K562 cells, CD69 and CD107a was not significantly up-regulated. These results demonstrated that T cell activation and degranulation needed to be stimulated by both target positive tumor cell lines.

### 3.6 Armored-T cells in combination with Rituximab enhanced *in vivo* treatment of hematoma

**Figure 5:**
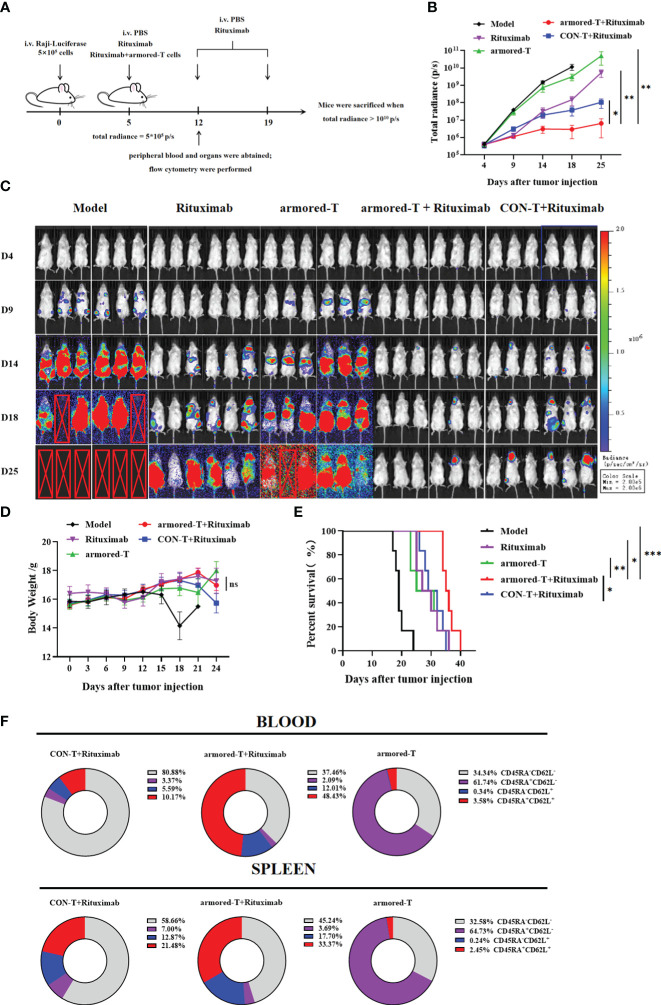
armored-T cells conjugated with antibody drugs exerted significant therapeutic effects on the Raji-Luciferase mouse model. **(A)** Grouping and treatment strategy of the Raji-Luciferase NCG model. The NCG mice were intravenously injected with 5×10^5^ tumor cells, and drug administration was once a week for two consecutive times, as soon as the total radiance reached 5×10^5^ p/s. **(B, C)** Tumor inoculation in mice, injection of D-luciferin potassium salt for *in vivo* imaging to determine tumor progression, and statistical analysis of total radiance. **(D)** Weight changes in mice every three days during the progression of the disease. The endpoint of survival **(E)** was set as a weight change ≥20% or when the total radiance reached 10^10^ p/s, and the mice were euthanized. **(F)** T cell differentiation status in blood and spleen samples one week after drug administration. Statistical significance was calculated using two-way ANOVA for multiple comparisons, the Kruskal-Wallis test for total radiance, and the survival period of mice was analyzed using the Log-rank test method. ns(no significance) p>0.05; *p<0.05;**p< 0.01;***p<0.001. Error bars represent standard error of the mean.

The Raji-Luciferase model was established by intravenous injection of Rituximab (30 mg/kg), armored-T cells, or co-incubation products of armored-T cells with Rituximab when total radiance reached 5×10^5^ p/sec/cm^2^/sr (**Figure 5A**). The results showed that survival was significantly longer in the armored-T cells + Rituximab group (**Figure 5E**), and total radiance was lower in this group than in the Rituximab and the armored-T groups (**Figures 5B, C**). However, there were no significant differences in body weight among all treated groups (**Figure 5D**).

Some mice were sacrificed one week after treatment in order to determine the distribution and retention of armored-T *in vivo* (**Figure 5A**). Peripheral blood and spleen were used to assay *in vivo* expression of armored-T. The expression level of FcγRI was basically consistent with armored-T group, and there was no downregulation of FcγRI molecules (**Supplementary Figures 6A, B**). Furthermore, it was evident that the proportions of armored-T+Rituximab group’s CD8+ T cells was higher than CON-T+Rituximab group in peripheral blood. This composition enabled effective elimination of tumor cells. Additionally, the spleen exhibited a similar ratio of CD4+ to CD8+ T cells, where the presence of CD4+ cells enhanced long-term tumor cell-killing capacity and sustained proliferation (**Supplementary Figures 6C, D**). Moreover, we analyzed the differentiation of T cells and the acquisition of effector-memory phenotypes (**Figure 5F**, **Supplementary Figure 6E**). In armored-T+Rituximab group, CD45RA+CD62L+ cells were generated in a large number.

### 3.7 Armored-T cells bound to Pertuzumab enhanced *in vivo* treatment of ovarian cancer

**Figure 6:**
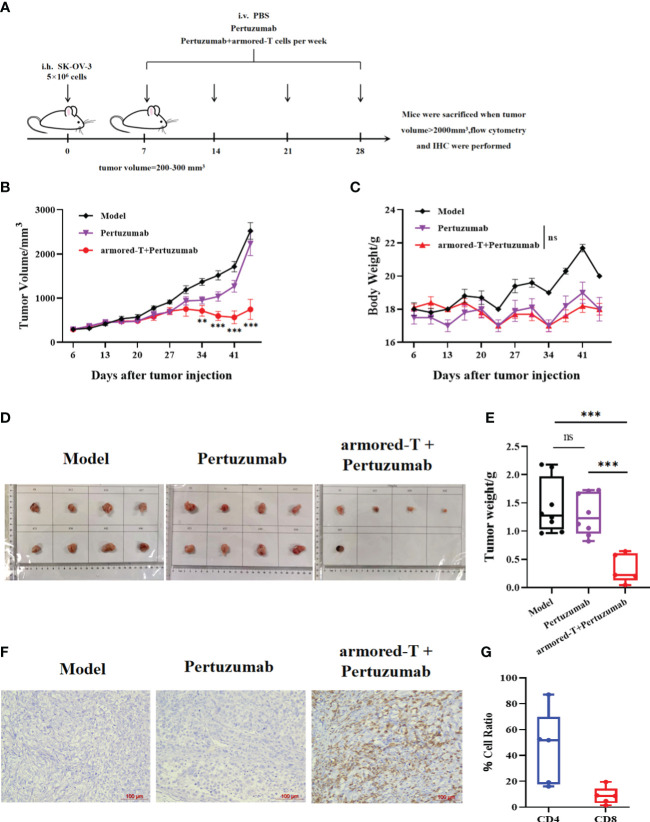
armored-T cells in combination with Pertuzumab resulted in effective tumor tissue infiltration in SK-OV-3 NCG mice. **(A)** After 5×10^6^ SK-OV-3 tumor cells were subcutaneously injected into NCG mice, the tumors reached approximately 200–300 mm^3^ on day 7. Then, the mice were divided into groups and treated with PBS, Pertuzumab, or armored-T cells-Pertuzumab conjugate *via* tail vein injection once a week for four consecutive weeks. During the treatment period, changes in mouse tumor volume **(B)** and body weight **(C)** were measured and analyzed. Photographs **(D)** and weights **(E)** of excised tumor tissues after the mice were euthanized. **(G)** CD4/CD8 ratio in peripheral blood samples taken from the periorbital region of mice. **(F)** Human T cell infiltration of excised tumor tissue samples, as determined using immunohistochemical analysis. Statistical significance was calculated using two-way ANOVA for multiple comparisons. ns(no significance) p>0.05;**p<0.01; ***p<0.001. Error bars represent standard error of the mean.

We established a HER-2+ SK-OV-3 ovarian cancer mouse model, and evaluated the therapeutic effect of Pertuzumab-bound armored-T cells by administering 2×10^6^ armored-T cells and Pertuzumab for four consecutive weeks.(**Figure 6A**). After 10 days of treatment, there were tumor inhibitory effects in the Pertuzumab group and the armored-T cells + Pertuzumab group, with better effect in armored-T+Pertuzumab group at about the 20th day (**Figure 6B**). At the late stage of treatment, the tumor volume of the armored-T+Pertuzumab group was significantly smaller than that of the Pertuzumab group, basically implying effective control of tumor growth and even regression (**Figures 6B, D**). It showed that drug combination had no effect on the growth status of mice because there were no difference in body weight between treatment groups (**Figure 6C**).

On day 39 after tumor injection, the mice were sacrificed and the tumor tissues were excised. The size and weight of tumor tissue in the armored-T+Pertuzumab group were significantly smaller than those in the Pertuzumab group and CON-T group (**Figures 6D, E**). This indicates that the combination effectively inhibited tumor growth. Analysis of T cell retention that at 18 days after armored-T injection, the T cells penetrated the tumor tissues and were retained for more than two weeks (**Figure 6F**). Moreover, the number of CD4+ T cells was higher than that of CD8+ T cells, which indicated the persistence of T cells (**Figure 6G**).

## 4 Discussion

For refractory tumors, we hope to transform life-threatening diseases into controllable and treatable chronic diseases. This means improving the Objective Response Rate (ORR) and progression-free survival period (PFS) for patients, while controlling Minimal Residual Disease (MRD) indicators, improving treatment efficacy and prognosis, and preventing recurrence. To achieve the above goals, it is necessary to overcome the challenges posed by the heterogeneity of malignant tumors and the complex TME. Currently, combination therapy has shown promising potential for development (16–18). A well-designed combination approach can enhance the ability of immune cells to infiltrate tumors, convert cold tumors into hot ones, and address the limitations of radiotherapy, chemotherapy, and monotherapy (19, 20). We hope to bring good news to patients who are incurable at the last line or who have failed in immunotherapy.

Currently, clinical studies primarily focus on the use of chimeric receptors of FcγRIII in T cells or NK cells (21, 22). However, the affinities of these receptors are not as high as that of FcγRI, and the clinical development is mainly focused on autoimmune diseases and hematologic malignancies (23). Based on these features, we selected high-affinity IgG molecules for addressing the efficacy problem in solid tumor treatment. Additionally, the combination strategy has the potential to overcome the issue of lack of ADCC-related receptors and to advance armored-T universal therapy by changing antibodies. Although we now have only tested four mAbs. In the future, we will combine different types of antibodies, such as bispecific antibody (bsAb) and antibody-drug conjugate (ADC), or the simultaneous combination of different target antibodies to produce effects similar to those of bsAb.

Our data highlight both the excellent killing effects and universal therapy of the combination strategy for addressing poor efficacy of antibodies and insufficient infiltration of immune cells. These results demonstrate the combined strategy is expected to turn cold tumor into hot tumor and make up for the deficiency of radiotherapy and chemotherapy. It has been suggested that CAR-T therapy may face the challenge of T cell exhaustion due to sustained activation (24). However, a clear advantage of the use of armored-T cells is that it ensures sustained activation of T cells while avoiding T cell exhaustion (25).

Based on these phenomena, we speculate the possible mechanism of T cell infiltration as follows: an interaction between T cells and antibodies forms a combination drug in a “plug-in” manner. The low pH in the TME can promote the embedding of antibody drugs into FcγR and inhibits their dissociation (13). Therefore, we think that the combination molecule has a longer “plug-and-play” time than mAbs, which delays their elimination from the blood and allows T cells to remain stable for a longer period of time. In order to further ensure the stability of the combination, we further proposed a pre-assembly strategy. This strategy enhanced the saturation of therapeutic antibodies on T cells, blocked the influence of autologous IgG, and enhanced the formation of immune synapse, thereby allowing immune cells to infiltrate tumor tissues. Compared with conventional treatment strategies, in which CAR-T cells and antibodies are administered separately (26, 27), our strategy may require a lower dose of CAR-T cells and the tumor is not easy to recur in the late stage (28, 29). These findings provide preliminary evidence for the potential therapeutic advantages of this combination strategy in the treatment of solid tumors.

At present, the high infusion dose of CAR-T products leads to long preparation time and high preparation cost. However, our combination strategy not only enabled a reduction in the dose and frequency of *in vivo* drug administration. The total amount of T cells used in treating ovarian mice was 8 × 10^6^ cells/mouse, which is significantly lower than the traditional dosage required for treating ovarian cancer in mice with HER-2-CAR-T (2 × 10^7^ cells/mouse) (30). This will help to reduce the pressure on production of lentiviruses and armored-T cells, cut the cost and improve the accessibility of cell therapy products. At the same time, the preparation time of armored-T cells can be shortened. This short-term process can preserve the stemness of T cells and increase their sensitivity to tumor response (31).

As we all know, CAR-T cell therapy lacks a suitable preclinical models to assess efficacy due to the complex immune environment of human body. Therefore, in the future, we need to further verify the efficacy of this combination therapy through Investigator-Initiated Clinical Trial (IIT).Now, we have completed chemical manufacture and control (CMC), and a fast and complete Good Manufacturing Practice of Medical Products (GMP) production line has been established. In the future, off-the-shelf will may be realized, which will benefit more patients.

In conclusion, the armored-T cells in combination with antibodies not only enhanced the efficacy of the antibodies, but also enabled T cells to perform ADCC. Moreover, this design eliminated the need for developing new molecules for different disease types: it simply requires combination of armored-T cells with the corresponding target-specific antibodies to achieve autologous T cell therapy. This is expected to lay the foundation for the universal treatment of CAR-T, while providing a new approach for antibody combination therapy in cancer treatment, thereby offering hope to patients with advanced cancer.

## Data availability statement

The raw data supporting the conclusions of this article will be made available by the authors, without undue reservation.

## Ethics statement

The animal study was approved by China Pharmaceutical University (approval number 2022-08-019) and by Nanjing Regenecore Biotechnology Co. Ltd. (approval number IACUC-2022-029). The study was conducted in accordance with the local legislation and institutional requirements.

## Author contributions

LT: Writing – original draft. QS: Writing – original draft. ML: Writing – original draft. XY: Writing – original draft. JM: Writing – original draft. YZ: Writing – original draft. YM: Writing – original draft. AZ: Writing – original draft. ZL: Writing – original draft. YL: Writing – original draft. XX: Writing – original draft. WG: Writing – review & editing.
