# Supplementary material for: Broadening anticancer spectrum by preprocessing and treatment of T- lymphocytes expressed FcγRI and monoclonal antibodies for refractory cancers
Source: Front Immunol. 2024 Jun 17;15:1400177. doi: 10.3389/fimmu.2024.1400177 (PMC11215118; doi:10.3389/fimmu.2024.1400177)
Supplement: Supplementary file 1 [file DataSheet_1.docx]

**Supplementary data**

**Supplementary Table1: list of abbreviations**

| **abbreviation** | **full name** |
| --- | --- |
| CAR-T | chimeric antigen receptor T |
| mAb | monoclonal antibody |
| TME | tumor microenvironment |
| FcγRI | Fc-gamma receptor I |
| ADCC | antibody-dependent cellular cytotoxicity |
| CDX | cell derived xenograft |
| FDA | Food and Drug Administration |
| DMEM | Dulbecco's Modified Eagle Medium |
| PBMC | Peripheral blood mononuclear cells |
| RIPA | radioimmunoprecipitation |
| SDS-PAGE | sodium dodecyl sulfate–polyacrylamide gel electrophoresis |
| PVDF | olyvinylidene fluoride |
| HRP | horse radish peroxide |
| CFSE | carboxyfluorescein diacetate succinimidyl ester |
| PI | propidium iodide |
| LDH | L-lactate dehydrogenase-release assay |
| ORR | Objective Response Rate |
| PFS | progression-free survival perio |
| MRD | Minimal Residual Disease |

**Supplementary Table2: Antibodies used for flow cytometry**

| Target | Fluorophore | Clone | Isotype | Source |
| --- | --- | --- | --- | --- |
| CD3 | PE | HIT3a | Mouse IgG2α, κ | BioLegend |
| CD3 | FITC | #001 | Mouse IgG2a | Sino Biological |
| CD64 | PE | 10.1 | Mouse IgG1, κ | BioLegend |
| CD4 | APC | RPA-T4 | Mouse IgG1, κ | BD Biosciences |
| CD8a | PE | RPA-T8 | Mouse IgG1, κ | BioLegend |
| Streptomycin | PE |  |  | eBioscience™ |
| CD20 | APC | 2H7 | Mouse IgG2b, κ | BioLegend |
| HER-2 | PE | 24D2 | Mouse IgG1, κ | BioLegend |
| CD69 | APC | FN50 | Mouse IgG1, κ | BioLegend |
| CD107a | FITC | H4A3 | Mouse IgG1, κ | BioLegend |
| IgG Fc | PE | QA19A42 | Mouse IgG1, κ | BioLegend |
| Fas | FITC | DX2 | Mouse IgG1, κ | BioLegend |
| FasL | PE | NOK-1 | Mouse IgG1, κ | BioLegend |
| CD45RA | APC | #27 | Mouse IgG1 | Sino Biological |
| CD62L | PE | #01 | Mouse IgG1 | Sino Biological |

**Supplementary Figure 1**: **Expression of FcγRⅠ-CAR in CD3^+^ human T cells.** **(A)**The figure shows gating strategy for armored-T.**(B)**The CD4/CD8 ratios of the armored -T and CON-T.

**Supplementary Figure 2**: **Positive rate and antibody binding efficiency of armored-J76. (B)** The ability to express of FcγRⅠ of J76 was detected by flow cytometry, and it’s gating strategy **(A)**. **(D)** The binding efficiencies of Rituximab and Pertuzumab after armored-J76/J76 cells were co-cultured with the antibody drugs for 1 hour at room temperature, as determined using flow cytometry, and it’s gating strategy **(C)**.

**Supplementary Figure 3**: **armored-T cells in combination with IgG antibody drugs exhibited enhanced cytotoxic activity and had good security. (A)** Status and aggregation of cells, as seen under the microscope after CD20^+^ tumor cells and CD20^+^ control cells were co-cultured with armored-T cells or CON-T cells and Rituximab (10 μg/mL) for 4 hours. The scale bar at the bottom right corner of the image represents a length of 20 micrometers. The region circled in red within the smaller circle indicates an enlarged portion of the image. **(B)**Tumor cells were co-incubated with armored-T/CON-T cells and mAbs (0/10 μg/mL) for 24 hours, and the emitted light were measured. **(C)** Release levels of IFN-γ in cell supernatants after 24 hours of co-incubation of Rituximab (10 μg/mL), armored-T/CON-T cells and Raji cells (E:T=3:1).**(D)** MCF-10A cells were co-incubated with armored-T/CON-T cells and Pertuzumab (0/10 μg/mL) for 4 hours, and the levels of released LDH were measured via peak absorption at a wavelength of 490nm. **(E)** The gating strategy for expressions of Fas and FasL in cells. Statistical significance was calculated using two-way ANOVA for multiple comparisons and t-test. **P* < 0.05; ***p* < 0.01; ****p* < 0.001. Error bars represent standard error of the mean.

**Supplementary Figure 4: Armored-T cells exhibited enhanced cytotoxic activity and cytokine secretion against tumor cells in combination with Nimotuzumab or Trastuzumab.** **(A)** Surface expression of EGFR on tumor cells, as assayed with flow cytometry. **(B, D)** Cytotoxicity in solid tumors, as assessed with LDH release assay. Tumor cells were co-incubated with armored-T cells (E:T=10:1) and Nimotuzumab (anti-EGFR) or Trastuzumab (anti-HER-2) (0.001-1 μg/mL) for 4 hours, and the released LDH levels were measured at peak absorption wavelength of 490nm. **(C, E)** Release levels of IL-2, TNF and IFN-γ in the cell supernatants after 24 hours of co-incubation of Nimotuzumab or Trastuzumab (0.01 μg/mL), armored-T cells or CON-T cells, and tumor cells (E:T=10:1). Statistical significance was calculated using two-way ANOVA for multiple comparisons. **P* < 0.05; ***p* < 0.01; ****p* < 0.001. Error bars represent standard error of the mean.

**Supplementary Figure 5**: The gating strategy for expressions of CD69 in T cells.

**Supplementary Figure 6**: **Phenotypic detection of T cells after one week in mice.** Persistence of armored-T cells in blood and spleen samples one week after drug administration **(B)** , as determined using flow cytometric analysis, as well as CD4/CD8 cell phenotyping **(D)**. The gating strategy for persistence of armored-T cells **(A)**, CD4/CD8 cell phenotyping**(C)**, and T cell differentiation status **(E)**. Statistical significance was calculated using two-way ANOVA for multiple comparisons. **P* < 0.05; ***p* < 0.01; ****p* < 0.001. Error bars represent standard error of the mean.
