# Supplementary figures and images for: Broadening anticancer spectrum by preprocessing and treatment of T- lymphocytes expressed FcγRI and monoclonal antibodies for refractory cancers
Source: Front Immunol. 2024 Jun 17;15:1400177. doi: 10.3389/fimmu.2024.1400177 (PMC11215118; doi:10.3389/fimmu.2024.1400177)

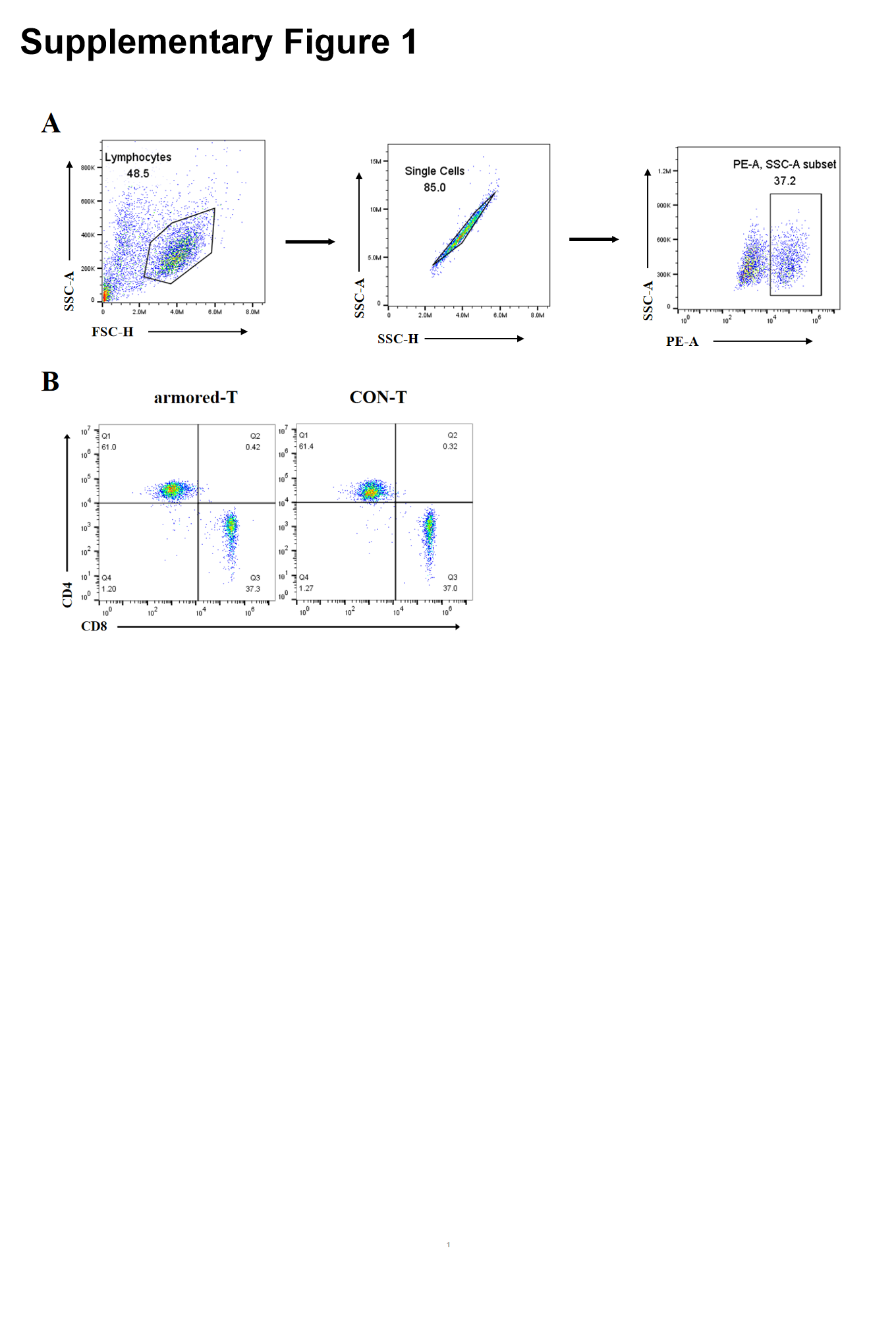

Supplement: Supplementary file 2 [file Image_1.tif]

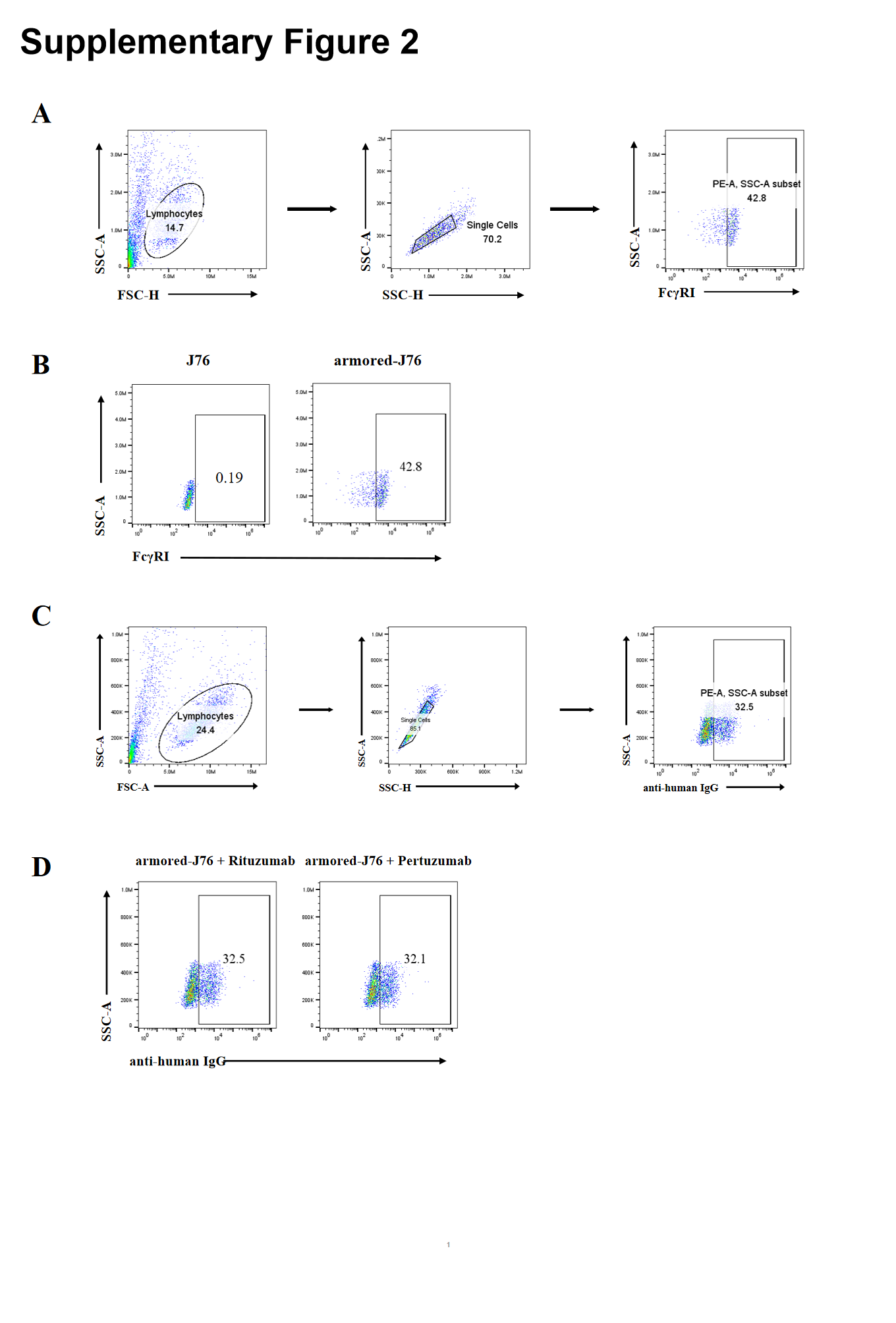

Supplement: Supplementary file 3 [file Image_2.tif]

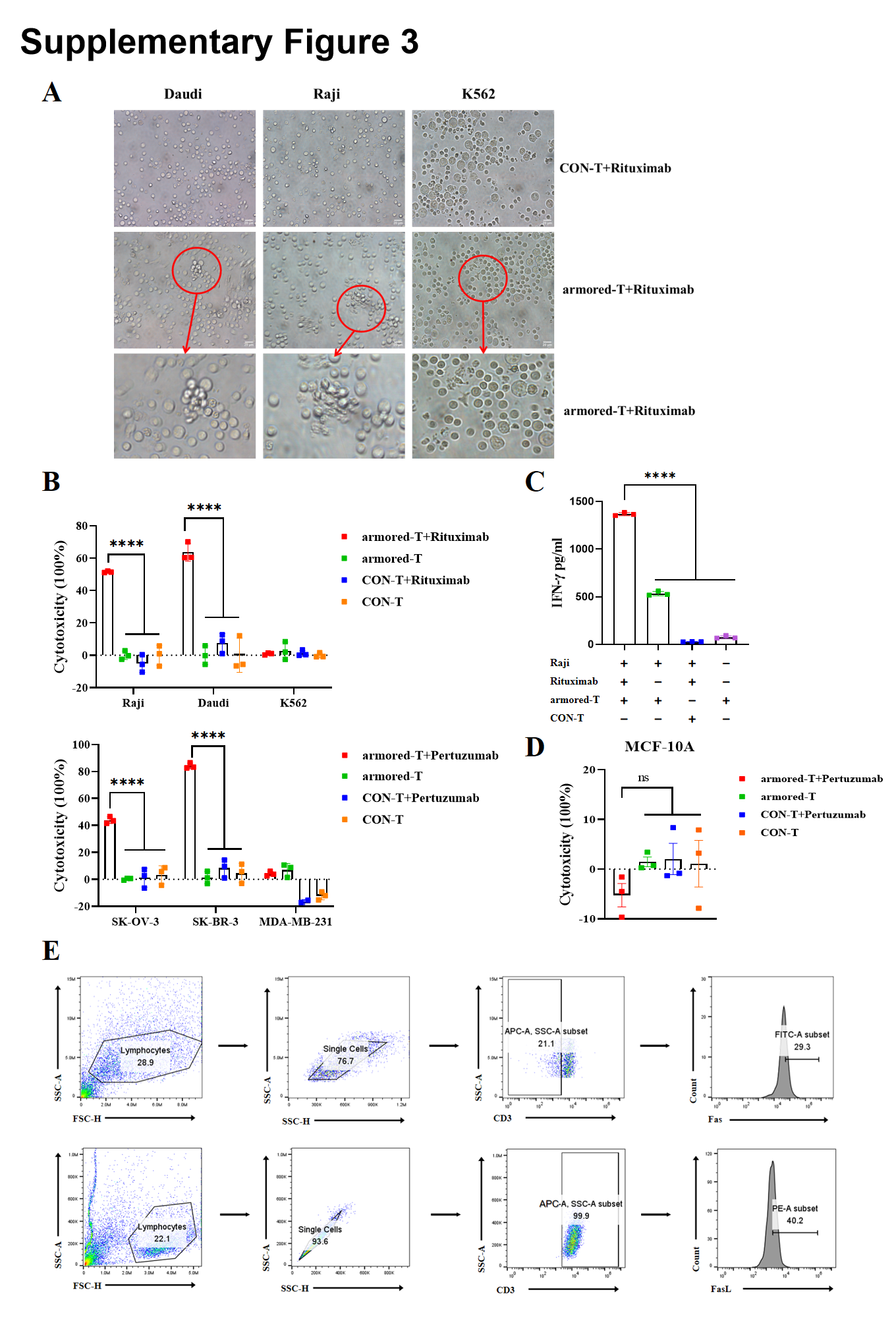

Supplement: Supplementary file 4 [file Image_3.tif]

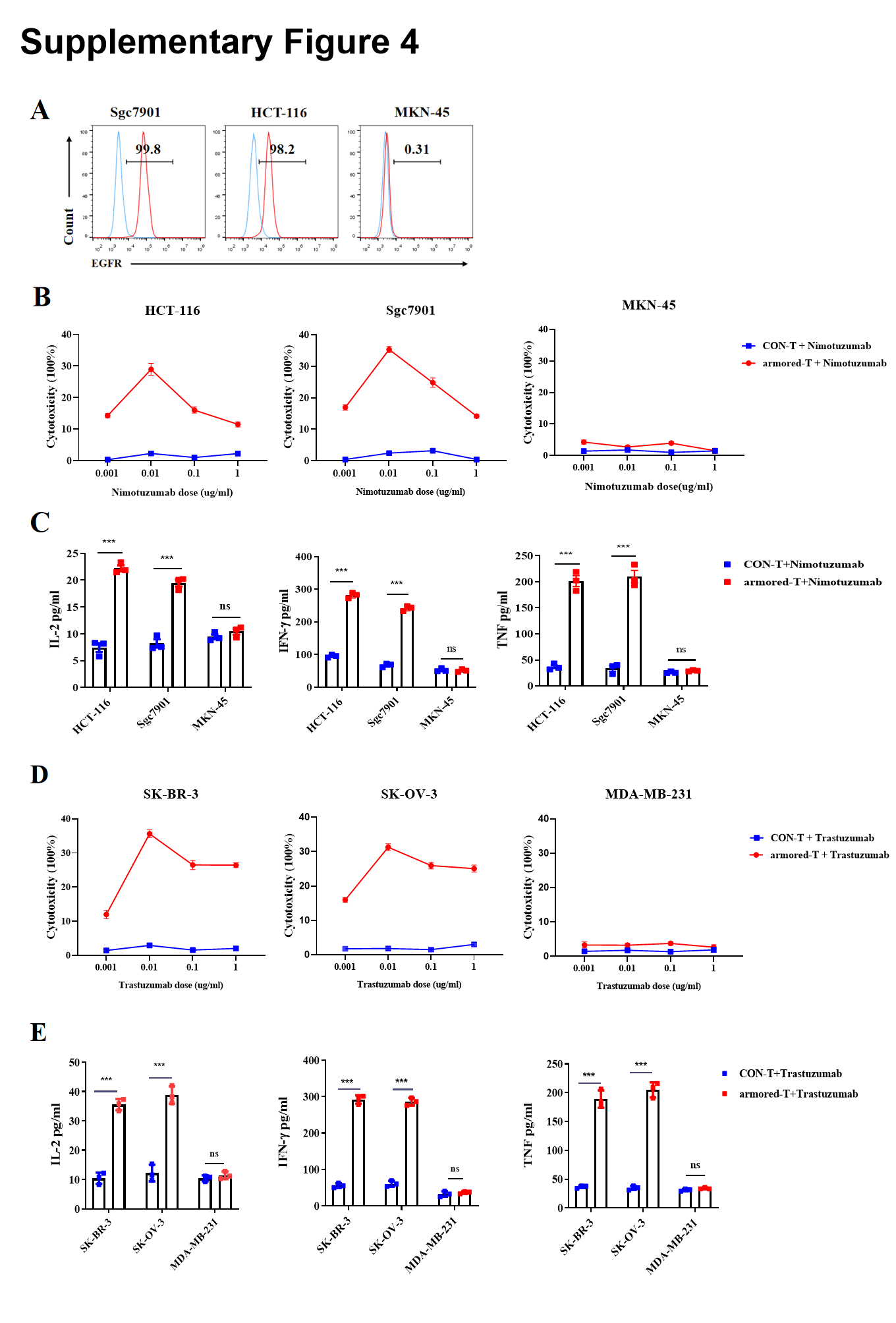

Supplement: Supplementary file 5 [file Image_4.tif]

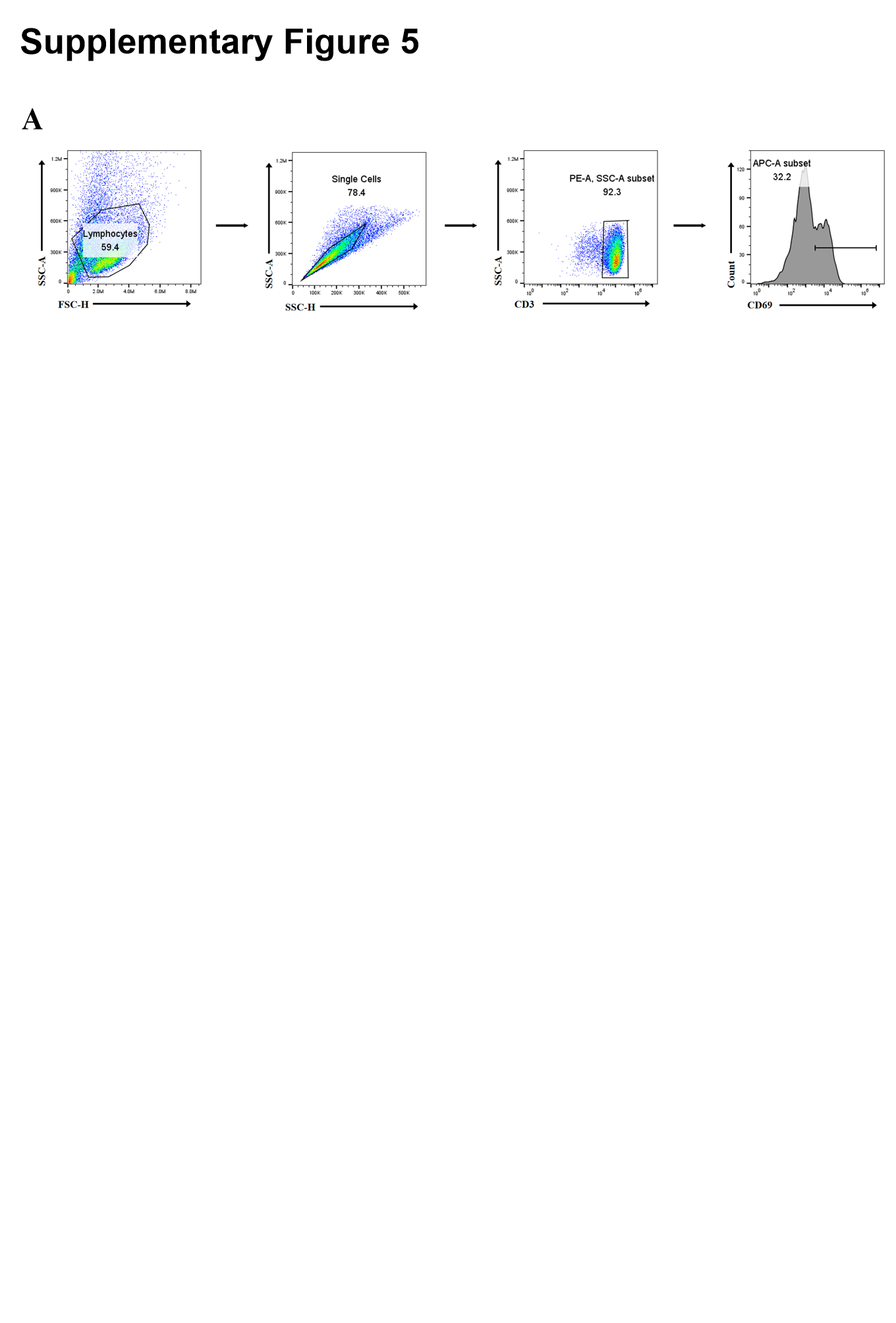

Supplement: Supplementary file 6 [file Image_5.tif]

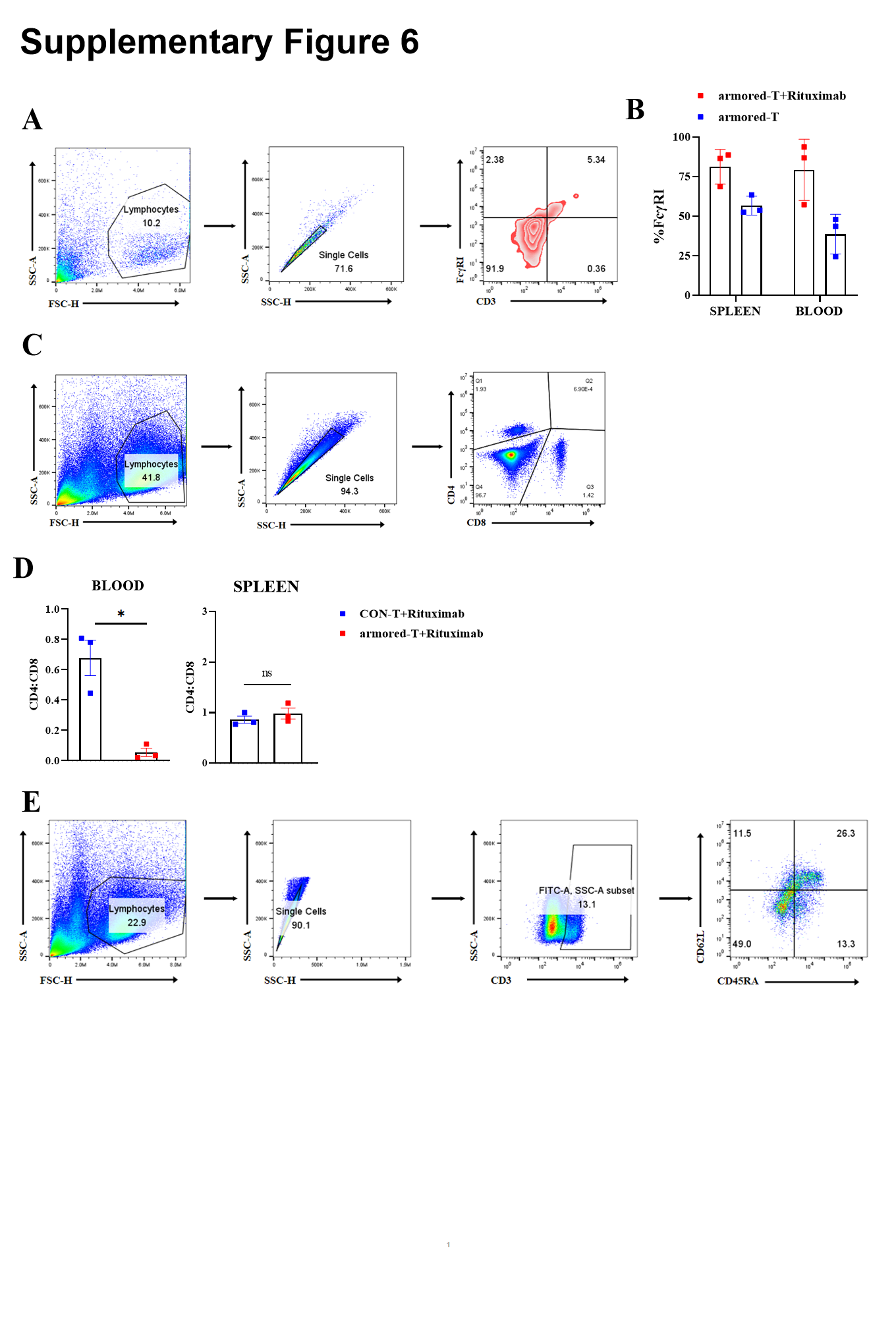

Supplement: Supplementary file 7 [file Image_6.tif]
